# Purification and Valorization of Waste Cotton Seed Oil as an Alternative Feedstock for Biodiesel Production

**DOI:** 10.3390/bioengineering7020041

**Published:** 2020-04-30

**Authors:** Djomdi, M. T. Leku, D. Djoulde, C. Delattre, P. Michaud

**Affiliations:** 1 Department of Renewable Energy, National Advanced School of Engineering of Maroua, University of Maroua, P.O. 46 Maroua, Cameroon; lekumitterand@gmail.com (M.T.L.); djoulde@gmail.com (D.D.); 2 Université Clermont Auvergne, CNRS, SIGMA Clermont, Institut Pascal, F-63000 Clermont-Ferrand, France; cedric.delattre@uca.fr (C.D.); philippe.michaud@uca.fr (P.M.)

**Keywords:** waste cotton seed oil, transesterification, biodiesel, purification, activated coal

## Abstract

This article is focused on the production of biodiesel from the waste cotton seed oil (WCSO), after purification, as an alternative to fossil fuels. Waste oil was collected from Sodecoton, a factory producing cotton seed oil in the Far North Cameroon. The WCSO was subjected to purification using activated coal, followed by transesterification under basic conditions (potassium hydroxide (KOH)), using methanol and ethanol. Some physico–chemical properties of biodiesel, such as absorbance of waste and purified oil, density, viscosity, water content, acid value, and its energy content were determined. The result of treating the WCSO with activated coal indicated that purification efficiency of activated coal increased with the contact time and the mass of the absorbent. Absorbance results directly proved that activated coal removed unwanted components. In the same way, activated coal concentration and exposure time influenced the level of free fatty acids of WCSO. The yield of methyl ester was 97%, while that of ethyl ester was 98%. The specific gravity at 25 °C was 0.945 ± 0.0601. An evaluation of the lower calorific value (PCI) was done in order to study the energy content of biodiesel. This was found to be a value of 37.02 ± 3.05 MJ/kg for methyl ester and 36.92 ± 7.20 MJ/kg for ethyl ester. WCSO constitutes feedstock for high volume, good quality, and sustainable production of biodiesel, as well as a realistic means of eliminating the pollution resulting from the indiscriminate disposal of waste oils from both household and industrial users.

## 1. Introduction

The global economy depends heavily on fossil energy generated from coal, petroleum, and natural gas. Increasing rise in cost and environmental concerns regarding the emissions of some particles linked to climate change (such as SOx, NOx, and CH_4_) that are released during fossil fuel production and utilization, are concerns that are taken into account in the development of alternate fuels like biodiesel from sustainable feedstock [1].

The world today is facing problems of energy supply shortage; as supply is always less than demand, due to population and industrial growth [2]. Countries face huge economic losses if the available energy in the country is not enough even for domestic use [3]. The world’s energy consumption according to the International Energy Agency (IEA) stood at 13.37 billion ton oil equivalent (btoe) in 2012 and 13.541 btoe in 2013, compared to just 6.1 btoe in 1973; the difference is clearly visible. Vegetable oil companies have increased in their numbers, thereby generating huge amount of waste. Sodecoton (an oil refinery at Maroua) is one of the oil-producing companies in the Far North Cameroon that consumes a great amount of energy both from hydro power and fossil fuel, but on the other hand, produces over 13 ton of waste oil every month. These large quantities of waste cotton seed oil are not valorized and constitutes a real problem for the company. These wastes cannot be easily burned, due to their high moisture content, and therefore, they are usually disposed of in landfills. If not handled properly, these large amounts of wastes can have harmful environmental effects [4].

Waste management is a major problem and a scientific challenge; in biofuel, for example, biogas is obtained from biomass in many homes today [1].

The question one might ask is, can this waste oil, which is available in large quantities in such an enterprise, not be used in the production of an alternative energy source? Could this enterprise use this energy to diversify its sources of energy? This could lead to a reduction in the dependence on fossil fuel as it can be used in its generators and boiler’s engines, especially during power failure.

This present study sought to adopt trans-esterification of Sodecoton’s waste oils as a reliable way to generate biodiesel, as well as curb the unregulated disposal and pollution caused by waste oils and, thus, evaluated the best reaction conditions for biodiesel production.

## 2. Materials and Methods

### 2.1. Waste Crude Cottonseed Oil (WCSO) and Chemicals

The WCSO was obtained from the Sodecoton Maroua company. It was stored at room temperature for seven days before the beginning of purification, analysis, and trans-esterification. All chemicals used (products of Sigma-Aldrich UK and J.T. Baker, Saint Louis, MO, USA) were of analytical reagent (AR) grade. Experiments were run in triplicate, i.e., each set of operation conditions was conducted three times, once with neat cotton seed oil used as a standard and the other two times with waste cotton seed oil (WCSO).

### 2.2. Purification of WCSO

Waste cotton seed oil was moderately heated up to 150–200 °C. This was done to remove water. Next, it was passed through muslin cloth with a pore size of 5–6 µm under vacuum to remove debris [5]. The filtrate was collected and stored at room temperature for better purification.

Laboratory bleaching was performed with different concentrations of activated coal (w/v) in a round bottom, three-necked flask of 500 mL, equipped with a thermometer and attached to a vacuum pump. Bleaching was conducted using an electromagnetic mixer with adjustable heater. The filtered waste oil was neutralized with suitable alkali. A total of 250 g of WCSO was stirred at 150 RPM under vacuum, using a vacuum pump, and was heated to 80 °C in a mantel heater. The vacuum was disconnected and the required amount of activated coal—0, 2, 4, and 8% (w/w)—was added to the hot WCSO. Then, the vacuum was applied, and the temperature was raised to 100 °C (±1). These conditions were kept constant for the desired time of 10, 20, and 30 min. Finally, the bleached oil was cooled to 70 °C and was allowed to settle. It was then filtered through filter paper (Whatman no. 541), under vacuum. A sample of WCSO was stored at 200 °C for further color development.

### 2.3. Determination of Free Fatty Acids (FFA) and WCSO Color

A sample of the cleaned WCSO was titrated against a standard solution in order to determine the concentration of free fatty acids (RCOOH) present in the waste vegetable oil [6]. The quantity (in moles) of base required to neutralize the acid was then calculated.

The color of purified WCSO was measured by UV–VIS spectrometer (Systronics, UV–VIS 118, India) to measure light absorbance in the visible region at 455 nm. This technique involves matching the color of light transmitted through a specified depth of oil with the color of light transmitted from the same source through a set of colored glass slides. Color reading is thus subjective and depends on the analytical skill judgment as well as on the type and model of colorimeter used.

### 2.4. Determination of Molecular Weights of WCSO

Gas chromatography/mass spectrometry (GC/MS) was used for waste cotton seed oil triglyceride analysis, through the identification of fatty acid profiles. Agilent technologies 7890A GC system USA (gas chromatography equipment) was used to separate oil triglycerides into fatty acid components. This involved three stages:

- Injecting a sample into the GC,
- Separating samples into constituent components,
- Detecting/identifying compounds present in the sample.

Oil samples were introduced at an initial oven temperature of 60 °C. The column temperature was programmed to increase to 200 °C at the rate of 10 °C min^−1^.

The injector and the flame ionization detector (FID) temperatures were set at 220 °C. The GC analysis of WCSO generated the fatty acid profiles and mass fraction of the fatty acids that constituted the oil triglycerides. The analysis of fatty acids was recorded as peaks on a chromatogram. The molecular weights of the biodiesel were determined.

### 2.5. Biodiesel Synthesis by Transesterification

The process of transesterification that leads to biodiesel production is highly dependent on the quality of raw materials, the amount of catalyst, and reaction temperature. The amount of free fatty acid calculated, indicate that the extra amount of catalysis will be added to the 3.5 g per liter of refined oil with an acid value less than one percent. 

**Figure 1:**
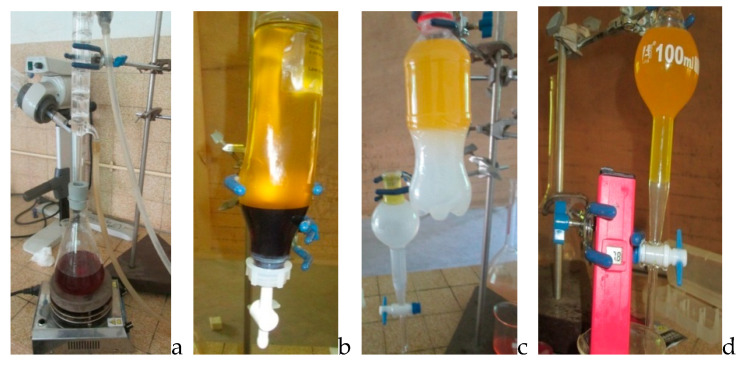

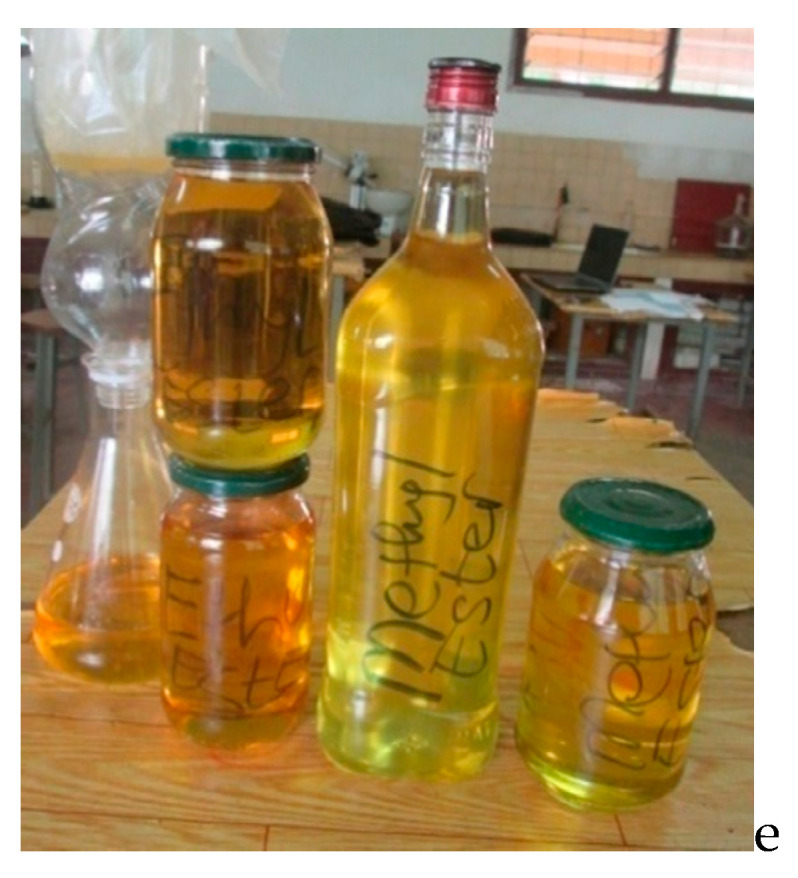
Biodiesel production process: (**a**) production, (**b**) separation, (**c**) and (**d**) washing, and (**e**) final products.

Next, 182 g of refined waste cotton seed oil acting as feedstock was weighed using a precision balance and put into a 500 mL flat bottom flask serving as a bioreactor, according to the methods of Andrew [3] and Xiaohu et al. [7]. This was heated to 60 °C on a magnetic stirrer heater. The flask was assembled with a condenser and the speed of the mechanical stirrer was adjusted to 700 rpm. In the meantime, 0.88% of KOH were mixed with 99% potassium hydroxide and used as catalyst, and considering the weight basis of cotton seed oil, was dissolved in pure methanol for the first batch and ethanol 95% for the second batch. The mixture was added to the reactor in a 6:1 methanol–oil molar ratio. The overall mixture was maintained at 60 °C on a magnetic heater. A stirrer was used to maintain the temperature and agitation rate of the reaction. A tap connected to a condenser was used to prevent loss of material (solvent, since it is volatile), for three hours. The products obtained were transferred to a separating funnel and allowed to stand for 24 h. The products separated into two distinct layers, a light yellow top layer (biodiesel) and a reddish brown bottom layer (glycerol). Biodiesels generated were cleaned for impurities, such as unconverted methanol, catalyst, soap, and traces of glycerol, by washing with several charges of warm distilled water, and were dried afterward at 120 °C in an oven for 30 min to eliminate residual moisture. Figure 1 below shows the different processes.

The clean methyl/ethyl ester was weighed in order to determine the water content and kept in an oven set at 105 °C for 48 h. This was to ensure that water that might have dissolved in the biodiesel was evaporated. The dried biodiesel was filtered using a 5 micron filter. The biodiesel was now set for other analysis. 

### 2.6. Calculation of the Yield of the Biodiesel

This is the proportion of the methyl or ethyl ester that was actually produced and is expressed as follows:

$$percentage\;yield\;of\;biodiesel=\frac{weight\;of\;biodiesel\;\left(g\right)}{weight\;of\;oil\;\left(g\right)}\times 100$$

### 2.7. Determination of Physico–Chemical Characteristics of the Biodiesel and Oil

The pure biodiesel and WCSO were characterized by ASTM (American Society of Mechanical Engineers). Different biodiesels produced under acidic and basic conditions were analyzed by ASTM standards, including acid value, saponification value, iodine value, moisture content, viscosity, refractive index, rancidity, peroxide value, soap content, specific gravity, density, pH, solidification rate, low calorific value, absorbance, and transmittance, using the same procedure as for the analysis of fat and oil samples.

The statistical analysis of all the data in this study were recorded in an Excel spreadsheet using Microsoft Office software version 2013, and the results were expressed as average ± standard deviation. The variation in the data collected and the statistical significance of the treatment effect were analyzed by analysis of variance. Statistical differences with a probability value less than 0.05 (*p* < 0.05) were considered significant. When the probability was greater than 0.05 (*p* > 0.05) the statistical differences were not significant.

## 3. Results

### 3.1. Purification of WCSO

In this work, the purification experiments were conducted with different masses of activated coal (0%, 2%, 4%, and 8%) at different contact times (10, 20, and 30 min). Results of the bleaching process with activated coal are reflected in Table 1. The process include decoloration of the oil, removal of majority of unnecessary soluble mineral ions, and great reduction in the density of the oil.

Table 1 shows that the absorbance values were decreased with respect to increased mass of activated coal (w/v in %). The absorbance value of purified WCSO decreased A = 0.83 to A = 0.21 at 455 nm; 2% (w/v) of activated coal improved the waste oil color and resulted in absorbance value of 0.83 within the contact time of 10 min. Further, the absorbance value was decreased to A = 0.71 and A = 0.67 within the contact time of 20 and 30 min. Additionally, 4% (w/v) of activated coal gave the results of absorbance value as A = 0.74, A = 0.55, and A = 0.40 within the contact time of 10, 20, and 30 min. Similarly, 8% (w/v) mass of activated coal purified the waste oil resulting in absorbance values of A = 0.32, A = 0.22, and A = 0.21 with contact times of 10, 20, and 30 min, respectively.

### 3.2. Fatty Acid Composition of Cotton Seed Oil

The composition of the oil extracted from cotton seed used in this work was found to be similar to that of oils extracted from other sources that have been used in biodiesel production (Table 2). Just like any other oil, cotton seed oil is made of fats and oils, which are made up of triglycerides: three molecules of fatty acids joined to a glycerol molecule. The chain length of the fatty acids and their organization on the glycerol backbone vary greatly, although in most of the edible oils it is with 16 and 18 carbons. Sample oils are a combination of fatty acids, both saturated (C14:0, 16:0), mostly palmitic acid (23.7%), and unsaturated (C 18:1, 18:2, 18:3), mainly constituted of linoleic acid (52.3%). Cotton seed oil is among the most unsaturated of the oils, others being safflower, corn, soybean, rapeseed, and sunflower seed oils. Cotton seed oil has a ratio of 2:1 of polyunsaturated to saturated fatty acids and generally consists of 65–75% unsaturated fatty acids including 18–24% monounsaturated (oleic), 42–52% polyunsaturated (linoleic), and 26–35% saturated (palmitic and stearic) [8]. Cotton seed is a non lauric acid oil. Table 2 gives a summary of the major fatty acids in cotton seed oil.

### 3.3. Physico–Chemical Properties of the Crude and Refined Oil

An evaluation of the properties of the waste crude oil and refined oil were made. The target was a good reduction of the acidity and free fatty acids levels of the crude oil. After the preparation it reduced to 0.1%, which is the norm required for edible oil. A summary of the results are presented in Table 3.

A very important aspect of the refined waste cotton seed oil is its very low peroxide value, which is an indication of the oxidation stability of the oil.

### 3.4. Biodiesel Production

The production of biodiesel was carried out varying different parameters such as catalyst concentration, different solvent (methanol and ethanol), and as well as solvent concentration.

### 3.5. Production of Biodiesel Based on the Variation of Catalysts

To achieve maximum yield of biodiesel using the waste feedstock, the optimum conditions were studied. The results of product yield of all experimental runs are summarized in Table 4.

The amount of biodiesel produced based on 182 g of oil was made using varied amounts of KOH as catalyst. Methanol solvent was used for the transesterification reaction. The reaction time was taken for 3 h. The temperature of the reaction was 60 °C and speed of the magnetic stirrer was stabilized at 250 rpm. The percentage of FFA content of the oil was 0.1%. The yield of the methyl ester was between 79% and 97%. The results of biodiesel yield obtained are presented in Table 4.

Another evaluation based on the solvent, but this time using ethanol instead of methanol, was carried out. The reaction time was fixed at 3 hours while the speed of the agitator was maintained at 250 rpm. Three sets of reactions were carried out and the results are shown in Table 5. The basic transesterification reaction of cottonseeds oil resulted in an average yield of 85.44% and 98.00% for 12:1 and 4:1 ethanol–oil ratios, respectively. These results confirm those of Xiaohu et al. [7] who also performed the transesterification of cottonseed oil under similar conditions and obtained a yield of 88%. This transesterification yield depends on ethanol–oil ratios and the amount of basic catalyst [3].

### 3.6. Post-Treatment of Biodiesel

The biodiesel after the reaction time was transferred into a separating funnel and allowed to decant for at least two hours for the glycerine phase to be separated from the ester phase. Then warm water at 50 °C was used to wash it. A 1% sulphuric acid solution was added to it during the second washing to neutralize it. The water was usually spread to reduce the possibility of formation of emulsions.

An evaluation of the lower calorific value of the ethyl ester and methyl esters were measured, and the results are presented in Table 6.

The calorific value of the biodiesel obtained from different oils used in this study showed that the calorific value of the obtained biodiesel is comparable to the results from the literature.

## 4. Discussion

The result of WCSO treated with activated coal indicates that purification efficiency of activated coal increased with contact time and mass of absorbent (Table 1). The absorbance results show that this parameter is linked to active coal concentration and time exposure. Thus, activated coal removes the unwanted plant pigments like gossypol, carotenoids, chlorophylls, etc. This is similar to the findings of Nandini and Sivasakthivel [9] and Toro Vazquez [10], who observed that carotenoids are efficiently removed by adsorption with activated carbon in soybean, cotton seed, corn, and squash seed oils. Similarly, groundnut oil has been decolorized by activated carbon from coconut shells. The adsorption of color bodies increased rapidly with an increase in temperature and mass of absorbents.

Table 1 shows that the high value of FFA (0.57%) is expected as the waste oil has not been purified. Activated coal concentration and exposure time influence the level of free fatty acids of WCSO. When increasing activated coal concentration from 2 to 8% at 10 to 30 min, the level of FFA began to be reduced between 0.44 to 0.17%, respectively. Many previous studies agreed that an excess of activated carbon increases waste oil purification significantly [9,11,12]. This pretreatment is important because under basic condition for transesterification, soap is formed, which interferes with the separation of glycerol. An emulsion with soap was formed, which also affected the yield of biodiesel because free fatty acid (FFA) rapidly reacted with the base to form soap. In basic transesterification, the formation of soap reduces the yield of biodiesel, but it is irreversible, and its rate is faster than the acidic transesterification [13].

Table 2 shows the fatty acid profiles of oils used in this study (%wt.) obtained by gas chromatography (GC).

The result shows the main characteristics of the methyl esters made from the fatty acids listed in the literature. When the unsaturated acids are hydrogenated, the melting temperature of the acid (or the oil of which the acid is a part) increases. This is an important consideration as far as using waste vegetable oils as feedstock for producing biodiesel is concerned. The more heavily used the oil is, the more hydrogenated it becomes, resulting in higher melting points for the molecules [14,15,16,17].

Best results were achieved with 6:1 methanol/oil ratio with 1.3 g catalyst followed by 3:1 ratio with 1.3 g catalyst. The yield percentage was affected drastically by reduction of the catalyst concentration under the same conditions. However, increasing both catalyst quantity and ratio had a negligible effect.

From the obtained results, the best percentage yield was obtained using the methanol/oil molar ratio of 4:1, potassium hydroxide as catalyst (1.2%), and a temperature of 60 °C (Table 5).

## 5. Conclusions

The present work aimed at valorizing waste cotton seed oil in the production of biodiesel, showing that activated coal improved the waste oil color and resulted in an absorbance value of 0.83 within the contact time of 10 min. Further, the absorbance value was decreased from 0.71 to 0.67 within the contact time of 20 and 30 min, respectively. Additionally, 4% (w/v) of activated coal resulted in absorbance values of 0.74, 0.55, and 0.40 within the contact time of 10, 20, and 30 min, respectively. Similarly, 8% (w/v) mass of activated coal purified the waste oil, resulting in absorbance values of 0.32, 0.22, and 0.21 with contact time of 10, 20, and 30 min, respectively. The cold neutralization technique was less energy consuming, but the quality and color of the oil was poor. The hot process gave oil of very good standards respecting international norms. A free fatty acid content of 0.1% and a color of 6.2 to 6.5 red were recorded from the hot process making it the best conditions for the process, since good quality biodiesel passes through good quality feed stock oil.

The refined oil was used in the production of biodiesel using methanol and ethanol as reagents while potassium hydroxide was used as catalyst. The highest yield 98% was obtained using ethanol while methanol gave a 97% yield. The fuel so far produced has lasted for more than three months without any deterioration, certifying the quality of the product. ASTM and other standards recommend fuel free of suspensions and here, the one produced has these properties. Biodiesel had a lower calorific value of 37.02 MJ/kg for methyl ester and 36.92 MJ/kg for the ethyl ester. The results are close to that of petroleum diesel of 42.50 MJ/kg. Thus, the company can finally subsidize and diversify its energy sources by the putting in place a biodiesel production plant. 

## Figures and Tables

**Table 1:** Purification parameters of waste cotton seed oil (WCSO) using activated coal.

Activated Coal % (w/v)	Temperature (°C)	Time (min)	Absorbance at 455 (nm)	FFA * (%)
0	100	10	1.87	0.56
		20	1.86	0.54
		30	1.87	0.57
2	100	10	0.83	0.44
		20	0.71	0.40
		30	0.67	0.38
4	100	10	0.74	0.40
		20	0.55	0.32
		30	0.40	0.29
8	100	10	0.32	0.25
		20	0.22	0.19
		30	0.21	0.17

* FFA, Free Fatty Acid.

**Table 2:** Fatty acids profiles of the cotton seed oil, WCSO, and PWCSO.

Fatty Acid (%)	Cottonseed Oil	WCSO **	PWCSO ***
Myristic (14:0)	1.0	0.9	0.7
Palmitic (16:0)	23.7	22.5	20.5
Palmitoleic (16:1)	0.6	0.5	0
Stearic (18:1)	3.4	5.5	4.5
Oleic (18:1)	19.4	52.1	48.0
Linoleic (18:2)	53.2	22.7	20.3
Linolenic (18:3)	0.5	0.3	0.3
Sum SFAs *	25	24	21
MUFAs *	22	57	48
PUFAs *	54	23	22

* MUFAs, monounsaturated fatty acids; PUFAs, polyunsaturated fatty acids; SFAs, saturated fatty acids. ** WCSO, waste cotton seed oil. *** PWCSO, purified waste cotton seed oil (activated coal 8%, 30 min).

**Table 3:** Physico–chemical properties of crude and refined waste cottonseed oil.

Property	Waste Crude Cottonseed Oil	Refined Cottonseed Oil
Physical state	Liquid	Liquid
Color	Black	6.2 red
Density g/L (28 °C)	0.92	0.91
Free fatty acid	8.178%	0.1%
Saponification value (mg KOH/g)	-	192
Peroxide value	-	2

**Table 4:** Variation of biodiesel yield basing on catalyst and methanol concentration.

Amount of Oil (g)	Catalyst Proportion (g)	Methanol–Oil Molar Ratio	Biodiesel Yield (%)
182	1.3	3:1	93.22
182	1.3	3:1	93.05
182	1.3	6:1	97.10
182	1.6	6:1	85.77
182	1.6	3:1	86.10
182	1.8	6:1	79.00

**Table 5:** Variation of ethanol–oil molar ratio.

Test	Sample	Catalyst (g)	Ethanol–Oil Molar Ratio	Ethanol (g)	Biodiesel Yield (%)
A	1	1.2	3:1	28.3	92.14 ± 2.01 ^b,c^
	2	1.2	4:1	37.75	98.00 ± 0.09 ^d^
	3	1.2	5:1	47.19	94.18 ± 0.18 ^c^
B	1	1.4	6:1	56.84	86.59 ± 2.13 ^a^
	2	1.4	9:1	81.20	89.34 ± 1.01 ^a,b^
	3	1.4	12:1	105.56	85.44 ± 1.42 ^a^
C	1	1.6	6:1	56.84	87.58 ± 0.93 ^a^
	2	1.6	9:1	81.20	90.44 ± 1.06 ^b^
	3	1.6	12:1	105.56	86.81 ± 2.83 ^a^
D	1	1.8	6:1	56.84	91.48 ± 0.47 ^b^
	2	1.8	9:1	81.20	94.50 ± 2.05 ^c^
	3	1.8	12:1	105.56	91.76 ± 0.27 ^b^

Numbers with the same superscript letters (a, b, c, d) on the column indicate that these values are not significantly different at *p* < 5%.

**Table 6:** Thermophysical parameters of product.

Weight of Biodiesel (g)	0.6412
Initial temperature (°C)	24.05
Final temperature (°C)	30.05
Change in temperature (°C)	6.45
Lower calorific value methyl ester (kcal/g)	8.84
Lower calorific value methyl ester (MJ/kg)	37.02
Lower calorific value of ethyl ester (kcal/g)	8.81
Lower calorific value of ethyl ester (MJ/kg)	36.92
